# Notes from Obstetric Practice

**Published:** 1887-07

**Authors:** Odo Betz

**Affiliations:** Heilbronn, Germany


### NOTES FROM OBSTETRIC PRACTICE.

*By Dr. Odo Betz, Heilbronn, Germany.*

For Daniel's Texas Medical Journal.

On the morning of the 14th of February, of the present year, I was called by a midwife to attend Mrs. B. I found her to be a small woman, of slender physique, thirty-six years old, and in labor with her third child. Her face was cyanotic and erythematous, her breathing labored and difficult. The midwife informed me that I had been summoned on account of prolapsus of the cord. Examining her, I found the cord prolapsed, but pulsating, the cervix relaxed, the os the size of a fifty cent piece, the right hand and shoulder presenting. Palpation showed the head to be on the left, and the breech on the right side. The fœtal pulsations 150 per minute. The history of the patient is as follows: She has never had any serious illness. Eight years ago she was delivered of her first child without assistance. It was a head presentation, but the child was stillborn. Six years ago the second child was born. This was also a head presentation, the child was born asphyxiated and could not be revived. In both instances the placenta showed signs of disease. For some time her legs have been somewhat swollen. I found the urine free from albumen, temperature normal, pulse 108, irregular, considerable dyspnœa. An examination of the lungs showed prolonged expiratory sound, sonorous and sibilant rales. Heart normal, pulsations weak and irregular. The midwife stated that the evening before the waters had broken, and that the patient had some pains, but none for the past few hours. I ordered an enema, and hot vaginal douches to stimulate the pains. After waiting a few hours till the os was well dilated, but the pains few and far between, and the patient suffering greatly from dyspnœa, I administered fifteen grains of powdered ergot, and immediately proceeded to turn the child by bringing down one foot, without using an anæsthetic, on account of the pulmonary difficulty. Ten minutes later, sharp pains set in, but simultaneously cessation of the fœtal pulsations, as shown by continuous auscultation. The child, a well formed girl, was then quietly extracted. but in an asphyxiated condition, auscultation showing cardiac pulsation, slower than normal. I employed Schulze's method of swinging the child, placed it in a warm bath, and poured cold water over it, etc., but could produce no effort at breathing. I then remembered that my father, Dr. F. Betz, had once saved a case of spasm oi the glottis by pouring compound spirits of ether into the nostrils, and concluded to try it in this case. Accordingly, a few drops were instilled into the infants nose, some applied externally to the cardiac region, the swinging continued, and I was overjoyed to see it attest an effort at breathing, which, after three-fourths of an hours work became regular, accompanied with crying.

In the meantime the placenta had been expelled spontaneously, an examination showing it to be a placenta marginata, otherwise normal. The mother made a good recovery, the lung trouble, œdema of the extremities and irregular pulse disappearing in course of a week. Antiseptic douches was the only treatment employed.

In itself there is nothing special or very unusual about this case, but I desire to call attention to a few points. While in attendance at the obstetric clinic in Tuebingen, have frequently witnessed with good results, the administration of ergot during labor to stimulate the pains. Prof. Satinger denies the occurrence of tetany of the uterus from ergot, and claims that he has never seen any evil results follow both as regards the mother or child. On the other hand, the late Prof. Schroder, in Berlin, urgently advised his students never to give ergot during confinement, except after the expulsion of the afterbirth, or immediately preceeding artificial delivery, claiming that ergot would produce dangerous tetanic contractions of the uterus. In my case I administered the ergot with a view to avoid dangerous hemorrhage from atony of the uterus following the delivery, the os being, however, widely dilated, and all the soft parts in a condition favoring immediate delivery. But I was soon convinced of the danger of ergot administered before delivery, when I found that the first few pains produced by ergot caused almost immediate stoppage of the fœtal pulsation. Recently there has been considerable discussion regarding the management of transverse presentations. In a debate on this subject, in the Berlin Gynæcological Society, Dr. Winters recommended immediate extraction after version, relating two hundred and thirty-eight cases, and only two per cent. of still born children, whereas, in eighteen cases where extraction was performed some time later after version, fifty per cent. of the children were dead. It should be remembered, however, that usually, when version is performed early, and extraction later, as in placenta prævia, etc., the life of the child is already greatly endangered.

There is no doubt that in my case it would have been better to have extracted the child immediately after performing version, but I was deterred from doing so on account of the great danger of post partem hemorrhage in the absence of all uterine pains.

Finally, I want to direct attention to the use of the compound spirits of ether applied to the nasal mucous membranes. It is certainly an excellent and popular cardiac stimulant, whether applied externally to the cardiac region, whether inhaled, or taken internally. It operates by dilating rhe capillaries of the cutaneous surface, diminishing the volume of blood in the heart, which then, having less work to do, reacts by a stronger impulse. In the asphyxia of the new born, by swinging the child, the chest expands, the ether evaporates and comes in direct contact with the bronchial mucous membrane, acting directly on the capillaries of the skin, and indirectly on the ganglia of the heart. Reflex inspiratory efforts are further caused by the local irritation of the ether on the nasal mucous membrane, frequently causing sneezing; the deep inspiration produced by this act followed by strong expiration. If this much is accomplished, by continuing artificial respiration, the asphyxia will soon be remedied.

